# Glymphatic system: an emerging therapeutic approach for neurological disorders

**DOI:** 10.3389/fnmol.2023.1138769

**Published:** 2023-07-06

**Authors:** Ying Gao, Kangding Liu, Jie Zhu

**Affiliations:** ^1^Department of Neurology, Neuroscience Centre, The First Hospital of Jilin University, Changchun, China; ^2^Department of Neurobiology, Care Sciences and Society, Karolinska Institute, Karolinska University Hospital, Solna, Sweden

**Keywords:** glymphatic system, AQP4, sleep architecture, neurological disorders, therapeutic approaches

## Abstract

The functions of the glymphatic system include clearance of the metabolic waste and modulation of the water transport in the brain, and it forms a brain-wide fluid network along with cerebrospinal fluid (CSF) and interstitial fluid (ISF). The glymphatic pathway consists of periarterial influx of CSF, astrocyte-mediated interchange between ISF and CSF supported by aquaporin-4 (AQP4) on the endfeet of astrocyte around the periarterioles, and perivenous efflux of CSF. Finally, CSF is absorbed by the arachnoid granules or flows into the cervical lymphatic vessels. There is growing evidence from animal experiments that the glymphatic system dysfunction is involved in many neurological disorders, such as Alzheimer’s disease, stroke, epilepsy, traumatic brain injury and meningitis. In this review, we summarize the latest progress on the glymphatic system and its driving factors, as well as changes in the glymphatic pathway in different neurological diseases. We significantly highlight the likely therapeutic approaches for glymphatic pathway in neurological diseases, and the importance of AQP4 and normal sleep architecture in this process.

## 1. Introduction

One of the most main characteristics of the central nervous system (CNS) is lack of the lymphatic system that plays an essential role in immune defense and homeostasis maintenance in the human body. The brain blood barrier (BBB) could compensate the former by restricting pathogens and other macromolecules such as antibodies in circulation entering into CNS. The scholars discovered the existence of lymphatic-like structures in the brain and their connection with cervical lymphatic node in the 19th century. In the past few decades, the BBB and blood cerebrospinal fluid (CSF) barrier (BCSFB) are considered as the main defender to maintain the homeostasis and clear the metabolic waste of CNS, until the discovery of glymphatic system in rodent in 2012 (Iliff et al., 2012). The glymphatic system concept states that the CSF is a directed flow, so that elements in CSF move with a speed exceeding the limit imposed by simple diffusion (Valnes et al., 2020). Subsequent studies have observed that the glymphatic system is also present in the human brain (Eide and Ringstad, 2015; Ringstad et al., 2017). One of the main characteristics of glymphatic system is the increased flow of CSF tracers in the sleep state and under anesthetic (Benveniste et al., 2017; Hablitz et al., 2019). This partly explains why sleep disturbance generally precedes the onset of cognitive decline and worsens clinical symptoms (Bah et al., 2019; Wang and Holtzman, 2020). Increasing researches over the past decade have shown that the glymphatic system acts as a ‘lymphatic system’ in clearing the metabolic waste and modulating water transport in the brain, and the dysfunction of the glymphatic system is proved to be involved in various neurological diseases through animal experiments, such as Alzheimer’s disease (AD), Parkinson’s disease (PD), epilepsy, stroke, traumatic brain injury (TBI), mood disorder and infectious or autoimmune disease in the CNS (Gaberel et al., 2014; Keir and Breen, 2020; Liu et al., 2021). Another clinical research showed that dysfunction of the glymphatic system might be associated with iron deposition in the normal aging brain (Zhou et al., 2020). Due to the BBB and BCSFB restrictions, many drugs do not show the same efficacy in the CNS as they do in the peripheral organs. In this review, we summarize recent research advances of the glymphatic system, including the anatomical structure and physiological function, driving factors and changes in the glymphatic system in neurological diseases, and the underlying therapeutic approaches. We also emphasize the role of aquaporin-4 (AQP4) and its changes in CNS disorders and relative potential treatment, and the importance of sleep architecture for glymphatic system function and neurological disease progress. Finally, we give our suggestions for future studies.

## 2. Structure and function of the glymphatic system

### 2.1. Structure of glymphatic system

**Figure 1:**
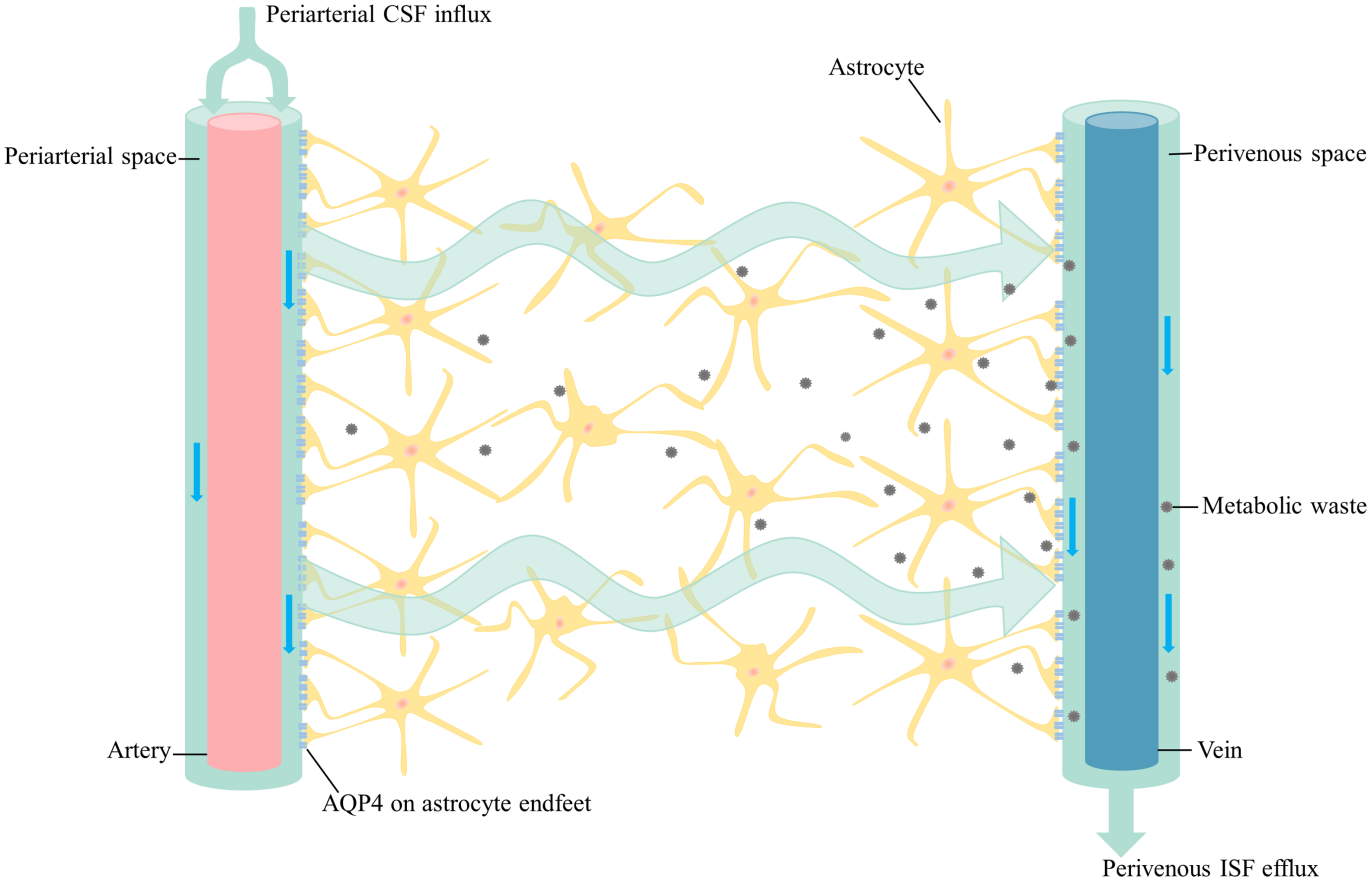
The glymphatic pathway. CSF from the subarachnoid space flows into the periarterial space along with the major arteries on the brain surface. CSF in the periarterial space is driven into the parenchyma by the pulsation of the arteries as it branches into the arterioles and capillaries. CSF enters the parenchyma with the support of AQP4, which then it mixes with ISF and drains metabolic waste from the parenchyma along perivenous space. In addition to the arachnoid granules, CSF exit sites include the olfactory nerve, cranial nerves, spinal nerves and meningeal lymphatics. CSF in the fossa cranial anterior flows into the cervical lymph nodes along with the olfactory nerve through lymphatic vessels of the nasal mucosa. Cranial and spinal nerves, as well as the meningeal lymphatics have been proved to carry CSF toward the cervical lymph nodes. CSF, cerebrospinal fluid; AQP4, aquaporin-4; ISF, interstitial fluid.

In addition to the traditional circulating pathway of CSF, there is another flow mode of CSF circulation, the glymphatic system, which is a structurally distinct fluid transport system that takes advantage of the perivascular space created by the outermost wall of the blood vessel and the vascular endfeet of astrocytes (Iliff et al., 2012). Part of CSF from subarachnoid space flows into the brain along the perivascular spaces (PVS) of arteries and pervades into brain parenchyma along with the arterioles, capillaries and venule (Iliff et al., 2012). CSF in the PVS of arterioles or capillaries mixes with interstitial fluid (ISF) and drains metabolic waste from the brain along perivenous spaces. This process is mediated by AQP4 on the astrocyte endfeet facing the perivascular space, which has a polarized expression on astrocytes (Benveniste et al., 2019b; Keir and Breen, 2020; Figure 1). The waste-carrying CSF in the perivenous space then leaves the brain via the meningeal lymphatics and deep cervical lymphatic vasculature, as well as the cranial and spinal nerves (Aspelund et al., 2015; Louveau et al., 2015). From the axial point of view, the innermost layer is the outer wall of the vessels and the outermost layer is the end-feet of astrocytes expressing abundant AQP4, which forms a cavity as the PVS fulfilled with the CSF (Mathiisen et al., 2010).

Until the discovery of the glymphatic system in 2012, it was believed that the recycling of its own protein waste was the primary clearing route for the metabolites of the brain (Rubinsztein, 2006). Only a small number of proteins are known to be transported across the BBB and are considered to degraded by the classical protein way, including autophagy and ubiquitination (Ballabh et al., 2004; Thibaudeau et al., 2018).

### 2.2. Probable function of glymphatic system

CNS metabolic waste was previously thought to be cleared by cellular degradation and slow diffusion or active transport by the BBB (Tarasoff-Conway et al., 2015). Recent advances in the study of the glymphatic system through animal models have been remarkable with the development of *in vivo* imaging techniques. Iliff et al. (2012) used real-time two-photon imaging techniques to delineate a brain-wide fluid system that relies on polarized expression of AQP4 and is functionally similar to the peripheral lymphatic system, in which they named as glial-lymphatic or glymphatic system. Over the past decade, the glymphatic system has been suggested to have a significant effect on brain waste clearance and intracranial pressure balance maintenance. The currently great interest of the glymphatic system is that the glymphatic system has been proved to have crucial effect on protein clearance, such as amyloid β (Aβ), α-synuclein and other proteins, which are involved in the pathogenesis of AD, PD and other neurodegenerative diseases (Iliff et al., 2012; Jucker and Walker, 2013; Xu et al., 2015; Da Mesquita et al., 2018). Interchange between ISF and CSF promotes the clearance of metabolic waste in the brain (Louveau et al., 2017). Besides the waste clearance, the glymphatic system is involved in the modulation of intracranial pressure and the transport of excess interstitial fluid in the brain (Ringstad et al., 2017; Nedergaard and Goldman, 2020). Based on the high density of astrocyte-related lipoproteins and lipid transporters, the glymphatic system is also contributing to the lipid transport and glucose supply (Rangroo Thrane et al., 2013; Lundgaard et al., 2015). Recent work found that fluid stress opened N-methyl-D-aspartic acid (NMDA) receptors in astrocytes, increasing calcium current, suggesting that the glymphatic system might play a role in signal transduction (Plog and Nedergaard, 2018), which may a potential direction in future research.

### 2.3. Relations among glymphatic system, the BBB, and meningeal lymphatic system

The role of cervical lymph nodes in the CSF flow has been observed in the 19th century. Researchers found that there are paravascular pathways in experimental hydrocephalus that go beyond the classical CSF pathway. At the same time, cervical lymph nodes and pia mater were also found to be involved in CSF reflux (Zhang et al., 1990). In the recent years, Aspelund et al. (2015) described the structure and function of the meningeal lymph system in 2015. They found a network of lymphatic vessels in the dura mater that drain CSF from the adjacent subarachnoid space and brain ISF via the glymphatic system (Aspelund et al., 2015). Finally, these lymph vessels transported fluid to the deep cervical lymph nodes via foramina at the base of the skull (Aspelund et al., 2015). A growing body of evidence has revealed that meningeal lymphatics have key effects on metabolite clearance, immune surveillance, and glymphatic flow out of the brain (Alves de Lima et al., 2020; Ding et al., 2021; Graham and Mellinghoff, 2021). Surgical ligation of the cervical lymphatic vessels prevented the flow of tracers into the deep cervical lymph nodes and the accumulation of tracers in the meningeal lymphatic vessels. In the capillary cross section, the BBB is composed of endothelial cells, pericytes, basement membrane and astrocytes from the inside to the outside (Lv et al., 2021). The structural anatomy of BBB and the glymphatic system partially overlaps, but how they interact is unclear in detail. Astrocytes participate in both the BBB function and glymphatic flow. Previous clinical study has demonstrated that enlarged PVS in the basal ganglia is associated with a higher BBB leakage rate, supporting the possibility that the dysfunction of glymphatic flow is a risk factor for BBB disruption (Li et al., 2019).

In summary, the BBB is a restricted structure between the parenchyma and circulating blood, which regulates the entry of blood nutrients into the brain, defends brain tissue from toxic components of the blood, participates in the metabolic efflux of the CNS. The glymphatic pathway is a convective system in which CSF originates in the arachnoid space, passes through the parenchyma and finally ends at the deep cervical lymph nodes. The most remarkable function of the glymphatic system that has yet been accepted is the clearance of metabolic waste in the brain. Meningeal lymphatics are primarily found in the dura mater and are one of the efflux pathways of the glymphatic system.

### 2.4. Expression of AQP4 in glymphatic system

AQP4 is the most highly expressed AQP in the mammalian brain. Except the supraoptic nucleus and the subfornical organ, which AQP4 is uniformly expressed in the astrocyte membrane. In the other domain of the brain, AQP4 is highly concentrated in astrocytic endfeet enwrapping the cerebral blood vessels (Nielsen et al., 1997; MacAulay, 2021). An early quantitative immunogold analysis on retinal macroglia indicated that glial endfeet contain a 10-fold higher density of AQP4 than non-endfeet membranes (Nagelhus et al., 1998). One study, through a survey of 123 healthy participants, showed that the AQP4-gene harbored an 8-SNP haplotype associated with AQP4 expression. The AQP4-haplotype is associated with a distinct modulation of slow waves in non-rapid eye movement stage (NREM) and also with a modulation of subjective and objective responses to prolonged wakefulness (Rainey-Smith et al., 2018; Ulv Larsen et al., 2020). Burfeind et al. (2017) found that none of the five AQP4 SNPs were associated with rates of AD diagnosis, age at onset of dementia, histology of AD pathology, but they were associated with the cognitive decline progression in AD patients. The majority of AQP4 functions depend on the polarized expression. Another study demonstrated that perivascular AQP4 localization was significantly associated with AD status independent of age (Zeppenfeld et al., 2017), suggesting that not only the quantity but also the localization is necessary to maintain the function of the glymphatic system. AQP4 plays an essential role in brain water transport and waste clearance (Figure 1).

## 3. Driving factors of the glymphatic system

**Figure 2:**
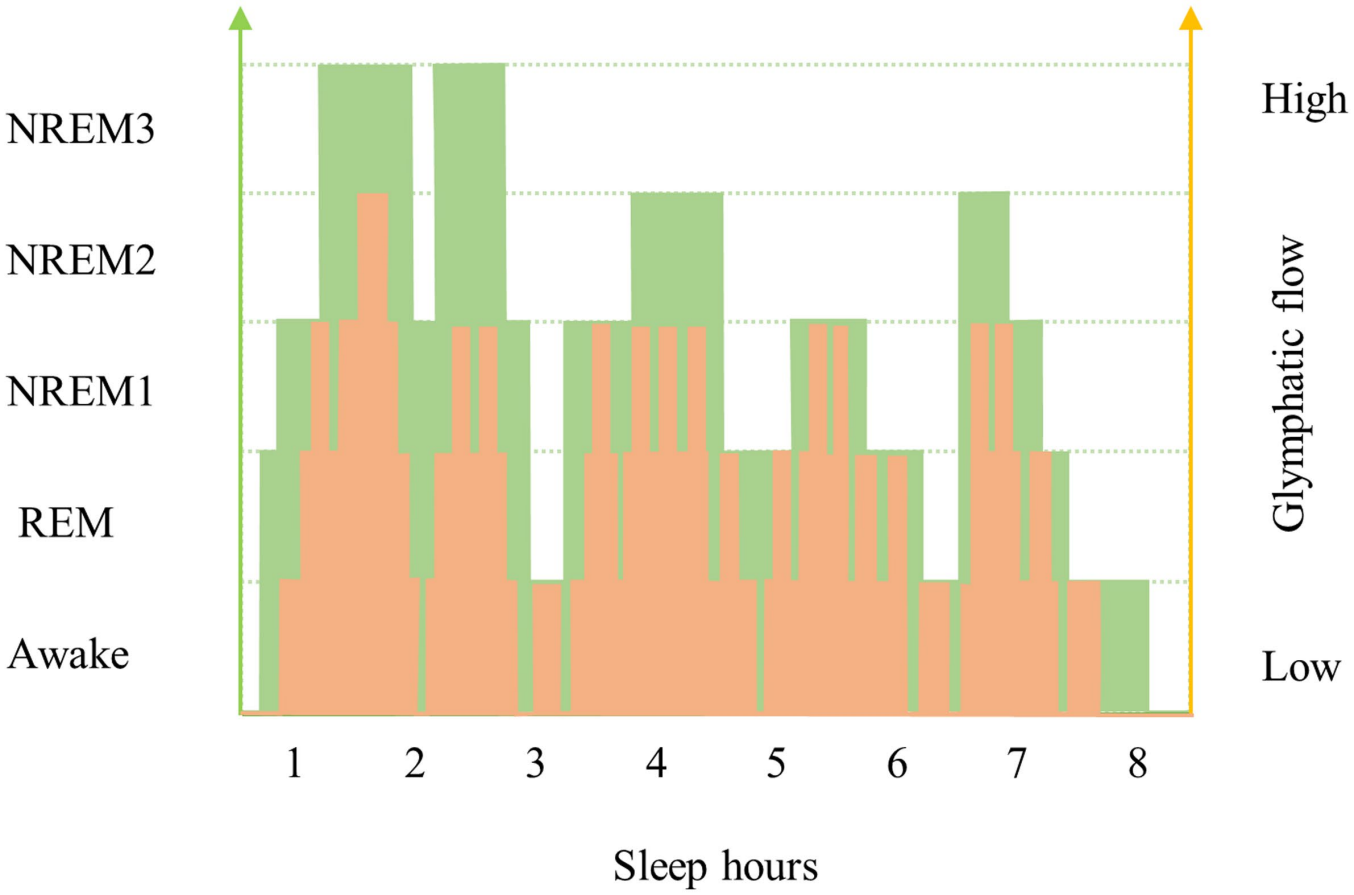
Changes in sleep architecture and glymphatic flow in young and old individuals. Sleep architecture changes with age, which is reflected by a decrease in sleep depth and an increase in waking frequency. One of the most significant causes of decreased glymphatic flow in older people is a change in sleep architecture, with lighter sleep depths and interrupted sleep stages. This figure shows the variation trend of the variability of the glymphatic clearing function between older and younger age groups. The green broken line and yellow broken line represent the sleep architecture and glymphatic flow speed of the young and the old, respectively. The area under the curve (AUC) reflects the accumulative waste removal of glymphatic system in the brain during sleep. As one gets older, the curve moves down and the AUC gets smaller, which means that the metabolic waste clearance of the brain decreases. This figure may also reflect the effect of pulsation on the function of the glymphatic system with age by replacing the sleep depth on the left axis with pulsation.

The fluid transport and waste clearance of the glymphatic system is affected by several factors (Plog and Nedergaard, 2018). It is commonly believed that the sleep–wake cycle may affect the glymphatic activity. Intact sleep architecture, which consists of NREM and rapid eye movement stage (REM), is the premise for the glymphatic activity. Based on the electroencephalographic (EEG) characteristics, NREM is classified as NREM 1 ~ NREM 3, which NREM 1 sleep is light, and NREM 3 sleep is the deepest sleep stage and is characterized by slow-wave (delta wave) EEG activity. During NREM3, the resistance to glymphatic flow is reduced. The velocity of glymphatic flow is strongly related to the depth stage of sleep. Glymphatic flow is highest during NREM 3 and decreases as sleep depth decreases (Figure 2). After sleep deprivation, subsequent NREM sleep was both longer and deeper than normal, and increased slow waves in the EEG were detected during recovery sleep (Hablitz et al., 2019; Nedergaard and Goldman, 2020). One human study provided *in vivo* evidence that one night of total sleep deprivation impaired clearance of the tracer from the human parenchyma. The results provide that the impaired of cerebral molecular clearance in the sleep-deprived group was not compensated by subsequent sleep over the next few days (Eide et al., 2021). Previous human study has shown that CSF influx increases at NREM 3, when nutrients and blood flow to the brain are reduced (Fultz et al., 2019). A recent study has shown that in mice, independent of the light–dark cycle, there is an increasing in glymphatic influx and clearance of small tracers from the brain during the day compared to the night, corresponding to a day-night variation in AQP4 localization (Hablitz et al., 2020). Another example illustrating the importance of normal sleep architecture for glymphatic function is the anti-anxiety and anti-insomnia medication, benzodiazepines, which reduce the stage of slow-wave activity during sleep (Lancel, 1999). Epidemiologic studies have revealed that there is an association between a higher exposure to benzodiazepines and dementia (Gomm et al., 2016; Tapiainen et al., 2018). This corresponds to the tendency between glymphatic clearance and sleep- wake cycles. This leads in a new direction to the desired replacement of benzodiazepines by new drugs with enhanced slow-wave activity to reduce the occurrence of dementia.

Glymphatic movement is also linked to the arterial pulsation and heart rate. A previous study of mice models showed that internal carotid artery ligation slowed the rate of perivascular CSF-ISF exchange, while dobutamine, an inotropic adrenergic agonist, increased the heart rate and the rate of paravascular CSF-ISF exchange (Iliff et al., 2013). It is well known that cardiovascular diseases could affect sleep quality. Previous studies have found that glymphatic function is suppressed in hypertensive rats (Mortensen et al., 2019; Xue et al., 2020). The ejection pressure of blood from the left ventricle is partially absorbed by the elastic arteries, causing the pulsations consistent with the cardiac cycles. Pulsations in the large arteries continuously transmit pressure waves along the major vessels. When the artery continues into the CSF-filled subarachnoid space, part of the ejection pressure is converted into kinetic energy of the CSF convection (Figure 1), thereby driving CSF into the parenchyma along the periarterial spaces (Iliff et al., 2012; Nedergaard and Goldman, 2020). Cardiovascular diseases associated with reduced cardiac output and arterial compliance, including congestive heart failure, atrial dysrhythmias, and hypertension, are thought to disrupt glymphatic function. Humberto et al. found that the pulsation of the arterial wall matched that the CSF flow rhythm, suggesting that arterial wall motion was the domain driving mechanism, via a process known as perivascular pumping. Increasing blood pressure does not alter the diameter of the artery, but changes the pulsations of the arterial walls, and thus reducing the net flow in the PVS (Mestre et al., 2018). All these findings demonstrate that arterial pulsation is a key driver of glymphatic flow.

Respiratory-related pulsatile cycles are another driving factor of glymphatic flow. Centripetal venous fluid flow, which increases with respiratory rhythm, may augment the venous space and drive glymphatic outflow (Kiviniemi et al., 2016). Body posture also affects the glymphatic flow, and the authors found that waste removal was most efficient at lateral position in the mice model (Lee et al., 2015).

Most of the driving factors discussed above are related to another force of the glymphatic flow, the aging. Sleep duration and depth, arterial pulsation and the respiratory pulsation cycles, all of these functions decline with age. As with many other disorders, glymphatic function reduced with aging. Compared with the young, clearance of intraparenchymally injected Aβ was impaired by 40% in the old mice. CSF-ISF exchange decreases with 27% reduction of intracortical arterioles and loss of polarized expression of AQP4 along the penetrating arteries (Kress et al., 2014). We speculated that the glymphatic flow declines with aging as many other disorders, leading to the accumulation of metabolic waste, which causes the occlusion in the glymphatic pathway and further deteriorates the glymphatic flow.

## 4. Changes of glymphatic system in different neurological disease

Previous studies have indicated that glymphatic flow and clearance activity decrease with aging and participate in a broad spectrum of diseases, especially those associated with sleep disturbance and neurological disorders with cognitive decline (Nedergaard and Goldman, 2020). In addition, it is involved in the pathology of other neurological diseases, such as epilepsy, and immune dysfunction. The glymphatic system participates in the process of a general and non-selective clearance route of toxic metabolites, and targeting glymphatic system fluid transport may contain novel therapeutic pathways for neurological disorders such as AD, PD, depression, epilepsy, cerebrovascular disease, infection and inflammation in the CNS (Xia et al., 2017; Liu et al., 2021; Zhang et al., 2021; Generoso et al., 2022).

### 4.1. Chronic neurological disorders

The dysfunction of the glymphatic system involved in animal models of chronic neurological diseases is primarily due to a decline in the clearance of waste proteins in the brain. Over the last decade, a growing number of studies have revealed the relation between AD and glymphatic dysfunction. Pathologically AD is characterized by the accumulation of Aβ plaques and neurofibrillary tangles of hyperphosphorylated protein tau (Xuan et al., 2022). Aβ, which plays a physiological role in synaptic regulation and neuronal survival, is degraded and cleared via multiple pathways, including phagocytosis, degradation, and drainage into the circulation through the BBB and glymphatic system (Zuroff et al., 2017; Reeves et al., 2020). Although the 75% drainage of Aβ depends on BBB transportation, recent studies have revealed that glymphatic flow plays a crucial part in the pathological process of AD. Pre-clinic research suggests that glymphatic pathway is a substantial factor in the clearance of Aβ (Iliff et al., 2012; Xu et al., 2015). In a mouse model of AD study, glymphatic failure significantly preceded Aβ deposits, which may be an early biomarker of AD (Peng et al., 2016). Recent mice study presented that CSF-ISF exchange and AQP4 polarization were impaired in tauopathy, the authors found that the use of TGN-020, a novel AQP4 inhibitor, could dramatically impair glymphatic CSF-ISF exchange and accelerate tau protein deposition in a mouse model. It demonstrated that AQP4 is not only plays a central role in the glymphatic system, but is also a novel target for the treatment of AD and other neurodegenerative diseases (Harrison et al., 2020). Previous radiological study of glymphatic system on AD patients found that the water diffusivity along perivascular spaces is positively correlated with mini mental state examinations (MMSE) score, which indicating that the glymphatic dysfunction is associated with AD severity (Taoka et al., 2017). Recent clinical study showed that the concentration of AQP4 in CSF was higher in neurodegenerative diseases compared with the subjects not affected by neurodegenerative diseases and AQP4 was positively corelated with total tau levels in CSF (Arighi et al., 2022). All discussed above predict that AQP4 may be a crucial factor in neurodegenerative disease in the future.

Age-related decline in CSF production, AQP4 polarization decreasing in astrocyte endfeet and Aβ aggregation all impede glymphatic flow and disturb waste clearance in the brain. The reduced clearance efficiency of the glymphatic system accelerates the plaque formation, which creates a vicious cycle in the progression of AD and deteriorates the exchange efficiency of CSF and ISF, as well as BBB transport (Peng et al., 2016; Xuan et al., 2022). One of the hallmarks of AD is disrupted sleep. One study in mice suggested that the glymphatic clearance of Aβ was double during sleep compared with the awake state (Xie et al., 2013). Another study in human revealed that sleep deprivation led to increasing Aβ level in parenchyma and interruption of NREM results in elevated CSF Aβ levels (Ju et al., 2017), which is consistent with the relation between sleep depth and the velocity of glymphatic flow. Additionally, amyloid plaques could accelerate cerebral amyloid angiopathy (CAA), and CAA promotes arteriosclerosis and reduces arterial pulsation, which further inhibits the clearance efficiency of glymphatic function (Weller et al., 1998; Peng et al., 2016).

Cerebral small vessel disease (CSVD), mainly featured as enlarged perivascular spaces and white matter hyperintensities, is a common instigator of dementia in the aging population. The pathological changes of CSVD are also presented as multiple atherosclerosis of the arteries, capillaries, and venules and cerebral amyloid angiopathy. The present research demonstrates that the glymphatic system plays a key role in the initiation and progression of SVD, and that both hypertension and diabetes participate in this process (Benveniste and Nedergaard, 2022).

Previous study on human has demonstrated that the cognitive decline is associated with the expression of AQP4 and the sleep–wake cycle (Ulv Larsen et al., 2020). The authors found that AQP4-haplotype is associated with NREM slow wave activity, which is strongest during the early sleep phase and is mirrored by changes in sleepiness and response times during extended wakefulness. Further studies have found that patients with various AQP4 SNPs show varying levels of cognitive decline after diagnosis of AD, and the polarized localization of AQP4 in astrocytes is disrupted with AD but preserved in older people with intact cognitive function (Burfeind et al., 2017; Zeppenfeld et al., 2017). The deletion of AQP4 accelerated amyloid plaque formation but did not modify the related proteins involved in synthesis or degradation of Aβ, suggesting that deletion of AQP4 leads to reduction clearance of Aβ in the brain (Xu et al., 2015). These studies suggest that regulation of AQP4 could be a clue to treatment of AD in the future. Glymphatic dysfunction also has been shown to be involved in other neurodegenerative disease, such as amyotrophic lateral sclerosis (Hirose et al., 2021), Huntington disease (Wu et al., 2020), idiopathic normal pressure hydrocephalus (Bae et al., 2021), and multiple sclerosis (Fournier et al., 2019).

The identification of glymphatic function has been presented for only a decade, and a growing number of studies have shown that glymphatic dysfunction is participated in the occurrence of various diseases. More studies could focus on both the pathogenic mechanisms and therapeutic function of the glymphatic system in neurological disease in the future.

### 4.2. Acute neurological disorders

Glymphatic flow has been elucidated to be involved in the pathophysiology of many acute neurological diseases, especially stroke. Stroke, classified as ischemic and hemorrhagic stroke, is the second leading cause of death and the third leading cause of disability in the world (Feigin et al., 2017; Campbell et al., 2019).

Subarachnoid hemorrhage (SAH) is a typical example to illustrate the changes of glymphatic flow in the course of stroke. Following SAH, blood components, particularly fibrin and fibrinogen, flow into the subarachnoid space from the ruptured vessel and then into the PVS along with the CSF. The inflow of blood components increases the formed elements and viscosity of the CSF, which leads to occlusion of the PVS and dysfunction of the glymphatic system. The occlusion of the PVS reduces CSF influx and ISF clearance, ultimately worsening cerebral ischemia and edema (Goulay et al., 2017). A recent study of mice SAH model, established by injecting autologous blood into the cisterna magna, showed a decrease in the influx of fluorescent tracer into the parenchyma and drainage to the deep cervical lymph nodes. Moreover, SAH impairs the polarization of AQP4 in the astrocytes and induces the accumulation of tau protein and immune cells in a study of mice models (Pu et al., 2019). In a non-human primates model study of SAH, researchers found that the parenchymal CSF circulation was severely impaired by SAH, as described in the glymphatic system of rodents. They suggested that the impaired glymphatic flow was associated with the delayed cerebral ischemia, which is a severe complication of SAH (Goulay et al., 2017). Interestingly, in a rodent stroke model, the authors reported that glymphatic dysfunction after SAH could be improved by intracerebroventricular injection of tissue-type plasminogen activators (Gaberel et al., 2014), which may be a novel target for improving the delayed cerebral ischemia complications of SAH.

Intracerebral hemorrhage (ICH) is another subtype stroke generally accompanied with hypertension or diabetes. Studies about the relation between ICH and glymphatic function is limited. Most studies have focused on the role of dilated PVS in ICH. A study involving 1678 participants displayed that dilated PVS was an independent risk factor for ICH (Duperron et al., 2019). In addition, dilated basal ganglia PVS has been found to be a novel risk factor for oral anticoagulants associated with ICH (Best et al., 2020), and dilated PVS is thought to be associated with ICH recurrence (Raposo and Viswanathan, 2020). Several studies have showed that reduction in AQP4 expression may alleviate cerebral edema and astrocyte injury following ICH (Chen et al., 2020; Zhang et al., 2020). However, other studies have presented otherwise about AQP4 in ICH. Tang et al. found that AQP4 deletion exacerbated neurological deficits, including cerebral edema formation, BBB damage, and neuronal apoptosis (Tang et al., 2010). AQP4 knock-out led to larger hematoma volume and more severe BBB disruption in another study (Chu et al., 2020), and in a study of mice ICH model, the researchers found that AQP4 deletion increased apoptosis following ICH via the modulation of cytokines, especially TNF-α and IL-1β (Chu et al., 2014). It is unclear how the glymphatic system is involved in ICH pathology and what the role AQP4 plays in the ICH development, positive or negative? Further studies are needed to clarify the mechanisms of the glymphatic system in ICH and how AQP4 acts on the pathological factor of ICH.

Acute ischemic stroke affects millions of people each year. Findings from the previous studies suggest that the glymphatic system is involved in the process of post-stroke cerebral edema. Acute ischemic stroke impaired CSF inflow at 3 h after occlusion of the middle cerebral artery and recovery 24 h after spontaneous arterial recanalization (Gaberel et al., 2014). In another study, the authors found that the extracellular fluid in liquefactive necrosis was toxic to cortical and hippocampal neurons for at least 7 weeks following a stroke. Toxic molecules in the liquefactive necrosis may leak through the glial scar and be cleared by a combination of glymphatic flow and microglial endocytosis, and they believed that the mechanism of post-stroke neurodegeneration was that the glial scar could not protect normal brain tissue from the leakage of toxic molecules in the liquefactive necrosis (Zbesko et al., 2018). But there is no consensus on how glymphatic function changes, and what role the glymphatic system plays in the course of acute ischemic stroke. A recent mice model study revealed that diffuse ischemia drives CSF influx into the PVS, which is the primary cause of immediate edema after acute ischemic stroke (Mestre et al., 2020). CSF influx drives acute ischemic tissue swelling, a pathogenic process triggered by ischemia spreading depolarization with subsequent vasoconstriction. They showed that the spreading edema depends on AQP4 expression (Mestre et al., 2020).

Traumatic brain injury (TBI) is defined as an alteration in brain function, or additional evidence of brain pathology caused by an external force (Menon et al., 2010). In mice models, TBI impaired glymphatic function and disrupted polarized localization of AQP4. Glymphatic flow was reduced by ~60% and persisted for at least 1 month after TBI (Iliff et al., 2014). A recent study on mice has shown that the polarized expression of AQP4 on astrocytes was reversed after 1-12 h TBI. During this period, the abundance of AQP4 on the astrocytic membrane increased, but the polarized position of AQP4 on the astrocytic endfeet decreased. After 12 h, the polarized position of AQP4 was reduced, presenting a shift from the endfeet membrane to the cytomembrane (Lu et al., 2020). The importance of polarized expression of AQP4 for glymphatic flow is verified again. Additional animal studies have shown that genetic knockout of AQP4 exacerbates glymphatic dysfunction and promotes the development of neurofibrillary plaque and neurodegeneration after TBI by reducing the clearance of tau protein, glial fibrillary acidic protein, S100B, and neuron-specific enolase (Plog et al., 2015; Piantino et al., 2022).

The glymphatic system affects the outcome of TBI mainly through two mechanisms. First, the loss of polarized location of AQP4 after TBI suppresses waste clearance and glymphatic flow movement, which leads to increased intracranial pressure and may be related to postconcussive headaches. Second, previous studies have revealed that sleep disturbances are observed in 30–70% of patients with TBI (Collen et al., 2012; Ouellet et al., 2015). As discussed in the front section, sleep disturbance impairs the glymphatic function and results in the accumulation of metabolic waste in the parenchyma, which may be associated with post-traumatic dementia (Piantino et al., 2022). Modulation of sleep and drugs targeting to AQP4 may be new therapeutic strategies for TBI in the future.

In addition to stroke and TBI, glymphatic dysfunction is also involved in more acute neurological diseases. A study on mice found that cerebral edema after status epilepticus may lead to glymphatic dysfunction, which may be an important factor in the p-tau aggregation and the onset of neurocognitive impairment after status epilepticus. The authors also demonstrated a temporary increase in AQP4 expression and depolarization of AQP4 after a state of status epilepticus (Liu et al., 2021). Dysfunctional glymphatic system is also observed in pneumococcal meningitis. A recent study of mice models presents that pneumococcal meningitis results in glymphatic dysfunction. The authors demonstrated that accumulation of bacterial components in the CSF is associated with the disruption of the AQP4 due to a detachment of the astrocyte endfeet from the BBB vascular cells, but not the altered AQP4 expression (Generoso et al., 2022). Another study also emphasized the glymphatic system is a potential key player in bacterial meningitis (Oggioni and Koedel, 2022). Interestingly, glymphatic system dysfunction also participates in cluster headache (Kim et al., 2022). More and more studies have emphasized the importance of glymphatic function in different neurological diseases. As a widely distributed clearance system in the brain, the glymphatic system may affect many neurological disorders, providing a new direction for future study on disease mechanisms and treatments.

## 5. Drug delivery barriers and potential approaches

Drug concentrations in the CNS are markedly lower than those in the systemic circulation due to the restriction of biological barriers, including BBB and arachnoid barriers. The concentration of therapeutic antibodies in CSF under intravenous treatment is only 0.01–0.1% of that in the systemic circulation (St-Amour et al., 2013). Compared with the dose of anti-Aβ antibodies directly injected into the brain, intravenous dose is 1,000 times to reverse cognitive impairments similarly (Banks et al., 2007). Due to the frequent side effects of intraventricular injection, such as bleeding and CNS infection, the most commonly used administration to avoid the BBB is the lumbar intrathecal route, which closely corresponds to the intracisternal injection in animal studies of glymphatic system (Lohela et al., 2022). With the development of nanomaterials technology, implantable intrathecal nanoparticle drug delivery systems for the treatment of CNS diseases are on the rise (Bottros and Christo, 2014; Fowler et al., 2020). The continuous nanorelease system avoids the damage caused by repeated intrathecal injections, leading to a more effective and less painful treatment method for CNS disorders. It has been successfully used in the treatment of spasticity with baclofen, which is the only FDA-approved GABA_b_ agonist for the potential treatment of spasticity (Kent et al., 2020).

## 6. Therapeutic targets of glymphatic system for neurological disease

As discussed above, the glymphatic system is involved in many neurological diseases due to its clearing function, fluid transport and wide distribution. Therefore, one or more of the major functional components of the glymphatic system would be a pharmacological target for the treatment of neurological diseases. In this section, we discuss several potential therapeutic targets of the glymphatic system for the treatment of neurological diseases.

### 6.1. Modulation of sleep architecture

Slow-wave activity in the EEG is consistent with deep NREM, which is the optimal stage for glymphatic flow to clear metabolic waste. Previous study has shown a positive correlation between glymphatic influx and cortical delta power in EEG recordings and a negative correlation between beta power and heart rate (Hablitz et al., 2019). Subsequently, it was shown that slow oscillating neural activity precedes coupled waves of blood and CSF flow in the brain (Fultz et al., 2019). Natural sleep or anesthesia are associated with a 60% increase in the interstitial space, resulting in a striking increase in convective exchange of CSF with ISF, which increased the rate of Aβ clearance during sleep (Xie et al., 2013). The effect of anesthesia on the glymphatic system is an appropriate example to illustrate how sleep architecture changes glymphatic flow. Different choices of anesthetics agent result in different rates of glymphatic CSF influx and efflux (Benveniste et al., 2017; Hablitz et al., 2019; Benveniste et al., 2019a). Dexmedetomidine, a selective α_2_-adrengergic agonist routinely used for sedation and commonly with markedly extensive slow-wave activity in EEG, could elevate the MRI contrast agent clearance rate in rat (Benveniste et al., 2017). Compared to isoflurane-only, the combined administration of dexmedetomidine and isoflurane clearly promotes CSF influx and has been shown to correlate directly with delta power in EEG (Hablitz et al., 2019). In addition, changing the sleep architecture is not the only mechanism by which anesthetics promote glymphatic flow. Preclinical studies have shown that anesthetic at high concentrations may induce cerebral vasoconstriction, which leads to enlarged perivascular spaces and promotes CSF influx into the brain (Ganjoo et al., 1998). Therefore, we speculate that targeting to the sleep architecture modulation would be a novel therapeutic modality not only for the neurodegenerative disease but also beneficial for stroke, TBI and other diseases.

### 6.2. Modulation of AQP4

As one of the most important constituent elements of the glymphatic system, AQP4 is a potential therapeutic target for many neurological diseases. Both genetic modification and pharmacological inhibition of AQP4 expression have been shown to reduce brain edema and improve outcomes of ischemic stroke through animal experiments (Yao et al., 2015; Pirici et al., 2017). TGN-073, an AQP4 facilitator, has been shown to promote ISF circulation within the BBB (Huber et al., 2018). Consistent with this, overexpression of AQP4 in mice has been shown to accelerate edema and poor prognosis after water intoxication (Yang et al., 2008).

Although AQP4 is an important potential target for neurological diseases, there are still many points that must be considered. The first is that AQP4 is also expressed outside of the CNS, in the sarcolemma of skeletal muscle, the inner medullary collecting duct of the kidney, the parietal cells of the stomach, and the epithelium of the exocrine gland (Frigeri et al., 1995; Verkman et al., 2017). Therefore, targeting AQP4 on astrocytes while avoiding binding to peripheral AQP4 must be considered to avoid side effects. In addition to the difficulties of crossing BBB in CNS drug delivery, off-target binding is another common occurrence. The second is that AQP4 has two main isoforms, a longer M1 isoform and a shorter M23 isoform, which form two distinct tetramers, a homotetramer consisting of all M1 or M23 isoforms, and a hetero-tetramers consisting of a mixture of M1 and M23 (Verkman et al., 2017). Drug specificity to different tetramers is another point to consider.

### 6.3. Modulation of CSF flow in perivascular space

CSF from the subarachnoid space flows into the parenchyma via the PVS, providing a mode of drug delivery. A previous study demonstrated that the influx of tracers from the subarachnoid space into the brain parenchyma depends on molecular weight. They found that as the molecular weight decreased, more tracers entered the brain parenchyma (Iliff et al., 2012). The FITC-d2000 (size as 2000 kD) was confined in the perivascular space. TR-D3 (size as 3kD) was more widely distributed than FITC-d2000, which is mainly entered into PVS. The lower-molecular weight A594 (size as 759D) moved quickly into parenchyma and only a bit constricted in the PVS. Pizzo et al. (2018) found that solute entry into the brain is consistent with diffusive transport and exhibits a clear solute size dependence, and co-injection of mannitol significantly increasing the speed of IgG flow into PVS in a dose-dependent manner. They found that single-domain antibodies (lower molecular weight as ~16.8 kD) could easier enter into PVS compared with IgG (higher molecular weight as ~150 kD). Another way of regulating the PVS flow is the regulation of arterial pulsations and cardiac cycles. Enlarged PVS was a predisposing factor for degenerative impairment and was observed to be dilated in hypertension by MRI. Glymphatic transport is compromised in both chronic hypertension and AngII-induced acute hypertension on mice models, which is probably due to decrease of the arterial pulsation and dilated PVS (Mortensen et al., 2019). The association between glymphatic flow and hypertension may in part explain the association between vascular pathology and AD (Mortensen et al., 2019). Systemic administration of dobutamine, an adrenergic agonist, has been proven to increase the rate of perivascular CSF-ISF exchange via enhancing arterial pulsation and cardiac contractility (Iliff et al., 2013). It is also explained that cardiac failure potentially reduced glymphatic flow exchange and accelerated cognition decline. Epidemiological study also showed that patients with cardiovascular diseases (such as heart failure, hypertension and atrial fibrillation) have a higher risk of AD (Cermakova et al., 2015). Conventional opinion in cardiac insufficiency patients at high risk for AD has attributed it to hypoperfusion of the brain, but with more research on glymphatic function, weakened vascular pulsation is a new pathological mechanism. Therefore, the regulation of pulsations and cardiac cycles is a new perspective for the treatment of neurological diseases. Increasing plasma osmolarity transiently is another effective way to promote glymphatic flow without affecting consciousness (Plog et al., 2018). However, it is often accompanied by pontine myeloysis syndrome (Lohela et al., 2022).

### 6.4. Other strategies

Adrenergic drugs are another way to improve glymphatic flow. Acute focal ischemia induces cortical spreading depolarization, which is featured as an increase in extracellular K+ and a suppression of neural activity. Systemic administration of adrenergic receptor antagonism before or after ischemic stroke in mice models accelerates the normalization of extracellular K+, promotes recovery of neural activity, reduces infarct volume, conserves AQP4 polarized expression on astrocytes (Monai et al., 2019). The authors suggested that adrenergic inhibitors promoted the exchange between CSF and ISF, accelerated extracellular K+ clearance, and are a potential treatment for stroke (Monai et al., 2019). A retrospective cohort study suggested that the β-blockers as highly BBB permeable drugs reduce the risk of AD compared with low permeability drugs in the process of hypertension treatment (Beaman et al., 2022). They hypothesized that β-blockers slowed down the development of AD due to improving metabolic waste clearance in the brain (Beaman et al., 2022). Activation of β-receptors reduces extracellular space volume and expands astrocytic processes (Sherpa et al., 2016). The administration of adrenergic receptors drugs modulated not only the ISF-CSF exchange and astrocyte, but also the cardiac cycle and arteriole pulsation, and further researches are needed to prove the systemic function of β-blockers in glymphatic pathway. In addition to those presented above, there are several non-pharmacological interventions could promote the CSF flow to ISF, including body gestures, exercise, and excess intake of polyunsaturated fatty acids (Lee et al., 2015; von Holstein-Rathlou et al., 2018; Liu et al., 2020). High alcohol intake reduces CSF influx, but low doses of alcohol promote glymphatic function, and vagus nerve stimulation enhanced CSF entry into the brain (Lundgaard et al., 2018; Cheng et al., 2020).

## 7. Discussion

Due to the difficulties of CNS drug delivery into brain, the severity of CNS disease, and the increasing incidence of neurodegenerative diseases in aging populations, a growing number of researchers are paying significant attention to the CSF/ISF exchange and the transport of metabolic wastes in the CNS. The main role of CSF in the CNS was considered to be as a buffer system until the discovery of the glymphatic system. Over the past decade, the glymphatic system has been shown to be involved in the pathophysiological processes such as metabolic waste clearance, water transport, and intracranial pressure modulation in the CNS. The discovery of the glymphatic system provides a deeper understanding mechanism of CNS diseases. At the same time, the glymphatic system supplies a new direction for the treatment of neurological diseases. We have elaborately discussed the changes in the glymphatic system in neurological diseases and potential targets for treatments. There are still a few concerns. Firstly, the pia mater and endfeet-PVS are permeable to small molecules and restricted to large molecules, which necessitates the selection of the correct molecular size of the drug in studies of glymphatic drug delivery. Secondly, based on the characteristics of the glymphatic system, we speculate that drug treatment for CNS diseases focused on nighttime (natural sleep stage) or sedation is more efficient than the traditional daytime dosing, because the glymphatic system is in a strong flow state in natural sleep stage and sedation or use drugs with longer half-lives during the day. Finally, intrathecal and intraventricular injections have not been widely used in clinical practice, due to the risk of invasions and probable infection. With the development of nanomedicine technology, the clinical application of these two therapeutic approaches will be greatly promoted. We propose that the glymphatic pathway provides a novel high-efficiency drug delivery pathway for CNS diseases.

## Author contributions

YG conceived and prepared the manuscript. KL revised the manuscript and the figures. JZ helped to conceive and reviewed the manuscript. All authors read and approved the final manuscript.

## Funding

This work was supported by the project of The First Hospital of Jilin University, Changchun, Jilin, China.

## Conflict of interest

The authors declare that the research was conducted in the absence of any commercial or financial relationships that could be construed as a potential conflict of interest.

## Publisher’s note

All claims expressed in this article are solely those of the authors and do not necessarily represent those of their affiliated organizations, or those of the publisher, the editors and the reviewers. Any product that may be evaluated in this article, or claim that may be made by its manufacturer, is not guaranteed or endorsed by the publisher.
